# Supplementary material for: The Effectiveness of Nutritional Interventions Implemented through Lady Health Workers on the Reduction of Stunting in Children under 5 in Pakistan: The Difference-in-Difference Analysis
Source: Nutrients. 2024 Jul 5;16(13):2149. doi: 10.3390/nu16132149 (PMC11243699; doi:10.3390/nu16132149)
Supplement: Supplementary file 1 [file nutrients-16-02149-s001.zip › nutrients-3002386-supplementary.pdf]

	Supplementary Table 1: Variables used for calculating Wealth Quintiles
1	Main source of drinking water in household.
2	Type of toilet facilities used by household.
3	Main material of the floor
4	Main material of the roof
5	Main material of the walls
6	Rooms available to households for sleeping
7	Household has electricity connection
8	Household owns radio
9	Household owns television
10	Household has landline phone
11	Household has refrigerator
12	Household has almirah/cabinet
13	Household has chair
14	Household has room cooler
15	Household has air conditioner
16	Household has washing machine
17	Household has water pump
18	Household has bed.
19	Household has a clock
20	Household has a sofa
21	Household has a camera
22	Household has a sewing machine
23	Household has a computer
24	Household has internet connection
25	Household owns a watch
26	Household owns a mobile telephone
27	Household owns a bicycle
28	Household owns a motorcycle or scooter
29	Household owns an animal-drawn cart
30	Household owns a car, truck or bus
31	Household owns a tractor
32	Household member owns a boat with a motor
33	Household member owns a boat with a without motor
